# Meta‐analysis and Mendelian randomization: A review

**DOI:** 10.1002/jrsm.1346

**Published:** 2019-04-23

**Authors:** Jack Bowden, Michael V. Holmes

**Affiliations:** ^1^ Medical Research Council Integrative Epidemiology Unit University of Bristol Bristol UK; ^2^ Medical Research Council Population Health Research Unit University of Oxford Oxford UK; ^3^ Clinical Trial Service Unit and Epidemiological Studies Unit (CTSU), Nuffield Department of Population Health University of Oxford Oxford UK; ^4^ National Institute for Health Research Oxford Biomedical Research Centre Oxford University Hospital Oxford UK

**Keywords:** Mendelian randomization, meta‐analysis, pleiotropy, two‐sample summary data MR

## Abstract

Mendelian randomization (MR) uses genetic variants as instrumental variables to infer whether a risk factor causally affects a health outcome. Meta‐analysis has been used historically in MR to combine results from separate epidemiological studies, with each study using a small but select group of genetic variants. In recent years, it has been used to combine genome‐wide association study (GWAS) summary data for large numbers of genetic variants. Heterogeneity among the causal estimates obtained from multiple genetic variants points to a possible violation of the necessary instrumental variable assumptions. In this article, we provide a basic introduction to MR and the instrumental variable theory that it relies upon. We then describe how random effects models, meta‐regression, and robust regression are being used to test and adjust for heterogeneity in order to improve the rigor of the MR approach.

## 1 INTRODUCTION

The primary aim of observational epidemiology is to determine the root causes of illness, with the focus of many epidemiological analyses being to examine whether exposure to a particular risk factor modifies the severity, or the likelihood of developing, a disease. Causal conclusions are rarely justified following a traditional analysis, even when strong statistical associations are measured between an exposure and outcome, because it is never certain that all confounders of the association have been identified, measured, and appropriately adjusted for. Mendelian randomization (MR)^1^ offers an alternative way to probe the issue of causality in epidemiological research, by using additional genetic variants that are hypothesized to satisfy the instrumental variable (IV) assumptions.

**Figure 1:**
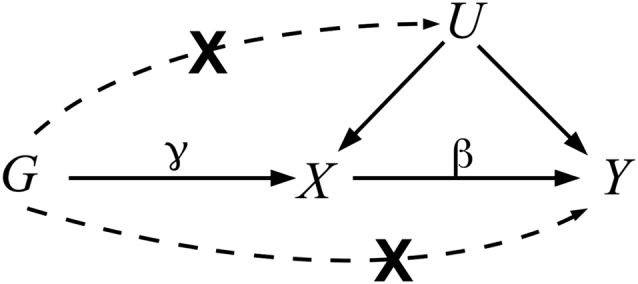
Causal directed acyclic graph (DAG) representing the hypothetical relationship between genetic variant G, exposure X, and outcome Y, in the presence of unobserved confounding, U. Solid arrows represent allowed relationships between the variables. Dashed lines represent relationships that are forbidden for G to qualify as a valid instrumental variable (IV). The G‐X and X‐Y arrows are parameterized by *γ* and *β*, with the latter denoting the causal effect of X on Y

Directed acyclic graphs (DAGs) are a useful tool, both to explain the rationale for an MR study and to clarify the IV assumptions that its validity rests on. Figure 1 shows a DAG relating the simplest single unit of genetic variation—a single nucleotide polymorphism (SNP) *G*—to an exposure, *X*, and outcome, *Y*, in the presence of unmeasured confounding, represented by *U*.

The true causal effect of the exposure on the outcome is denoted by the arrow from *X* to *Y* in Figure 1 and the parameter *β*. The “associational” estimate obtained from a simple regression of the outcome on the exposure could be systematically different from this causal effect, because confounding may be responsible for all, or part of, its magnitude. From the DAG in Figure 1, this can be understood by noting that the association between X and Y is contributed to by the direct effect path *X* → *Y* and the “back door” path *X* ← *U* → *Y*.^2^ Suppose, however, that a SNP *G* exists, which robustly predicts a proportion of the exposure that is unrelated to any confounders of the exposure‐outcome relationship. This is represented by the path *G* → *X* and the absence of a path between *G* and *U*. If, in addition, *G* can only influence the outcome through the exposure, as represented by the absence of a direct path from *G* to *Y*, then this SNP is said to be a “valid IV.”

G is usually coded as 0, 1, or 2 to reflect the number of exposure‐increasing alleles of a SNP an individual carries. This assumes that G exerts a linear per‐allele effect on X. The exposure itself is typically a continuous measure, for example, a person's blood pressure, body mass index, or cholesterol level. It will sometimes represent a binary health behavior, for example, whether an individual is a current smoker. The outcome of interest can be continuous but is often a binary variable, usually representing the presence or absence of a disease.

### 1.1 Testing for causality

If *G* is a valid IV, then any nonzero association between this SNP and the outcome provides evidence to support the hypothesis that the exposure causally effects the outcome and *β*≠ 0. This can be understood by noting that *G* is “d‐separated” from *Y* (or independent of *Y*) in Figure 1 upon removal of the path *X* → *Y*.^2^ Genetic variants have successfully been used to test for causality by looking at their association with the outcome in a variety of settings. However, most MR studies go further, by attempting to estimate the magnitude of the causal effect, *β*. Specifically, this parameter quantifies the effect on the outcome when the exposure is intervened on and changed by one unit, with all other factors remaining fixed.

## 2 QUANTIFYING THE CAUSAL EFFECT IN MR USING THE RATIO ESTIMATE AND TSLS

If SNP *G* is a valid IV, the exposure can be assumed to causally affect the outcome in a linear fashion with no effect modification, then the underlying SNP‐outcome association (denoted by Γ) should be the product of the underlying SNP‐exposure association (denoted by *γ*) and the causal effect of the exposure on the outcome, *β*. That is,

(1) Γ=βγ.

From Equation 1, the simplest estimate for *β* ( ${\hat{\beta }}_{R}$, where *R* stands for “ratio”) is obtained by dividing the SNP‐outcome association estimate by the SNP‐exposure association estimate to give:

(2) $${\hat{\beta }}_{R}=\frac{\hat{\Gamma }}{\hat{\gamma }}.$$

The standard error of the ratio estimate can be approximated via a Taylor series expansion of ${\hat{\beta }}_{R}$ using the delta method.^3^ The ratio estimate in (2) is calculated from two summary estimates, but it is also equivalent to the estimate obtained by the following two‐step procedure applied to individual level data on *Y*, *X*, and *G*:

Step 1 Regress the exposure on the SNP via the model:

(3) $$X|G=\gamma_{0}+\mathrm{\gamma G}+\varepsilon_{X}.$$

Step 2 Regress the outcome on the fitted values of the regression in step 1, $\hat{X}$, via the model:

(4) $$Y|\hat{X}=\beta_{0}+\beta \hat{X}+\varepsilon_{Y}$$

and report its estimated regression coefficient, $\hat{\beta }$. This is referred to as “two‐stage least squares” (TSLS).

When multiple SNPs are available as IVs, they can be easily incorporated into a TSLS analysis to yield a single causal estimate, by calculating fitted values based on a multivariable regression of the exposure on all SNPs together in model (3). In that case, *γ* and *G* would represent vectors of association parameters and SNP values for each individual. This automatically allows for any potential correlation between the SNPs, for example, due to linkage disequilibrium (LD). Standard errors for the TSLS estimate in (4) must take into account the uncertainty in the first stage model (3). This correction is performed as standard in most software packages.

Equation 1, the ratio estimate in (2) and the TSLS procedure in (3) and (4) are only strictly correct when Y is itself continuous. When Y is binary, logistic regression is typically used to quantify the G‐Y association in (2) or the association between the genetically predicted exposure and outcome in (4). In this case, the causal effect of a unit change in the exposure on the risk of Y will have a magnitude that depends on the reference level of X chosen (and so will not be constant). It will also be attenuated towards zero by an amount proportional to the residual variance in the logistic model not explained by $\hat{X}$. This is due to the noncollapsibility of the odds ratio.^4^ However, because genetic effects generally explain a very small amount of variation in the exposure, this means that the range of genetically predicted exposure levels is very narrow around the center of the distribution of X. Modeling the causal effect of moving between different levels of the genetically predicted exposure as a constant value therefore provides a very good approximation to the true “local” causal effect. For further discussion, see appendix 1 in Zhao et al.^5^

## 3 META‐ANALYZING MR ESTIMATES ACROSS STUDIES

Meta‐analysis has classically been used to combine MR estimates, derived using either the ratio or TSLS methods, across different epidemiological cohorts. Many of the pitfalls and challenges in synthesizing standard (ie, noncausal) estimates across studies are avoided in the MR setting, because the IV assumptions mean that confounder adjustment is unnecessary.

For example, the C‐reactive protein (CRP)–coronary heart disease (CHD) genetics collaboration^6^, ^7^ brought together 47 separate epidemiological studies to investigate the causal role of CRP, a marker of inflammation, on the risk of CHD, using individual level data on approximately 200 000 individuals.

**Figure 2:**
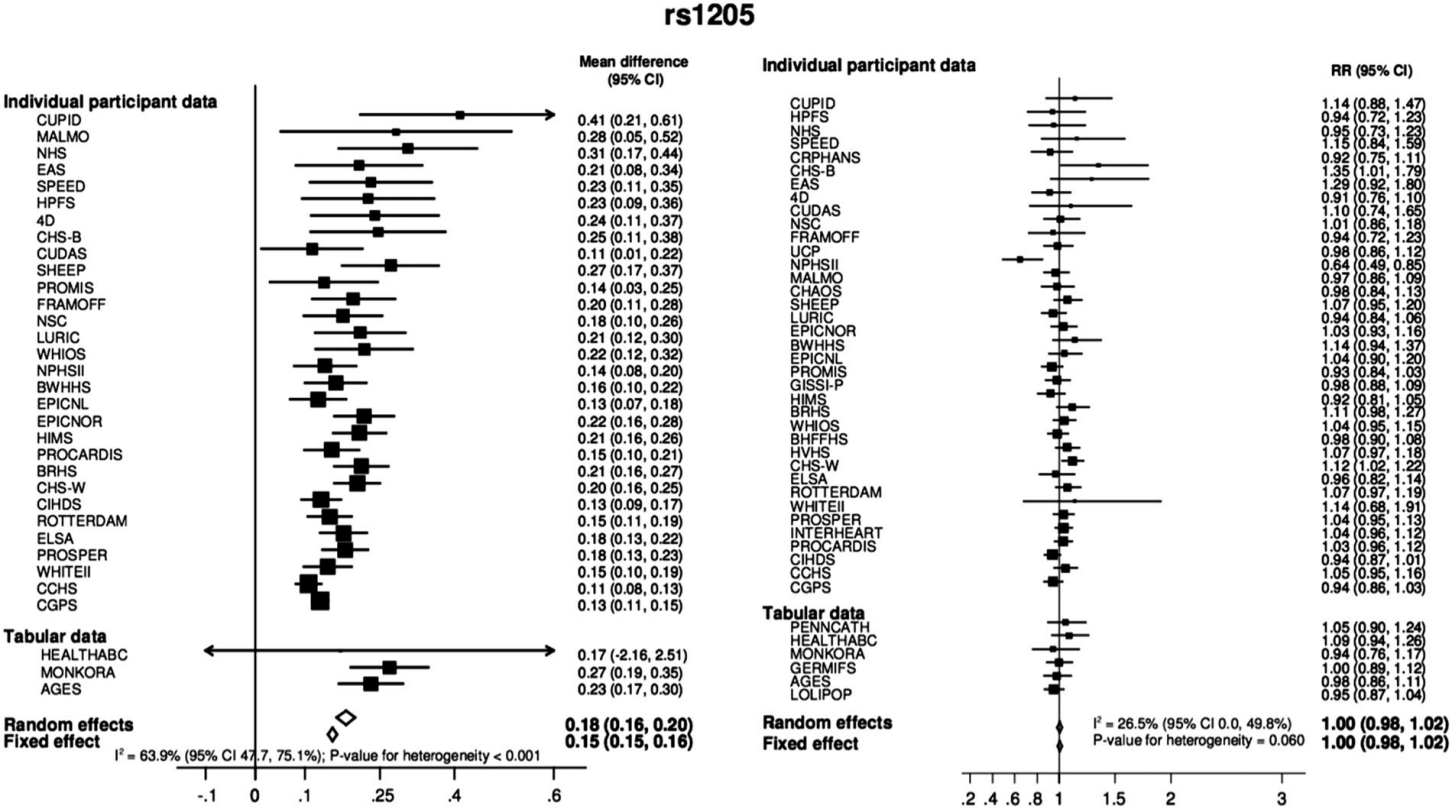
Meta‐analysis of the association of rs1205 with C‐reactive protein (left) and heart disease (right) in studies contributing towards the C‐reactive protein coronary heart disease genetics collaboration.^7^ Estimates reflect the mean difference in log CRP per allele (left) and odds ratio of CHD per allele (right). CHD, coronary heart disease; CRP, C‐reactive protein

Four SNPs located in the same gene region were utilized as IVs to predict circulating CRP levels. Estimates for the effect of each SNP on log CRP and CHD risk were derived for each study. The results were then meta‐analyzed across studies.^7^, ^8^ An example of the findings for one of the genetic variants (rs1205) is provided in Figure 2.

The collaboration's meta‐analysis provided important evidence in support of the emerging consensus that CRP is unlikely to have a major role in the development of CHD.

## 4 THE CHANGING FACE OF STUDY DESIGN: TWO‐SAMPLE SUMMARY DATA MR

The CRP example is typical of a traditional MR study design, in that it made use of individual level data and utilized a small number of correlated genetic variants with a known functional role on an exposure to firstly estimate study‐specific causal effects and secondly meta‐analyze the results across studies. Unfortunately, the level of cooperation and administrative burden required to share individual level data in this way has meant that this model is relatively inefficient for the large‐scale pursuit of MR analyses. In recent years, however, it has become possible in theory for anyone to conduct an MR analysis by combining summary estimates of SNP‐trait associations from two genome‐wide association studies (GWASs), released into the public domain by international disease consortia. This has become known as two‐sample summary data MR.^9^, ^10^

Specifically, suppose that a single common SNP is measured in two separate GWAS (eg, “studies 1 and 2”) where study 1 measured its association with trait *X* and study 2 measured its association with trait *Y*. A ratio estimate for the causal effect of *X* on *Y* can be obtained by dividing the SNP‐*Y* association estimate from study 2 by the SNP‐*X* association estimate in study 1, just as in formula (2).

Typically, GWASs report summary data estimates of associations with a trait for the strongest SNP within a specific genomic region, across many regions encompassing the entire genome. This has led to a dramatic increase in the number of uncorrelated variants that that can, in principle, be used within an MR analysis. Ratio estimates for each SNP are then combined to yield an overall causal effect using standard inverse variance weighted (IVW) meta‐analysis formulae:

(5) $${\hat{\beta }}_{\mathrm{IVW}}=\frac{\sum_{j}w_{j}{\hat{\beta }}_{\mathrm{Rj}}}{\sum_{j}w_{j}}.$$

Here, ${\hat{\beta }}_{\mathrm{Rj}}$ represents the ratio estimate obtained from the *j*th SNP, and *w*_*j*_ is its inverse variance weight. Traditionally, so‐called “first order” weights are used, which assume that the denominator of the ratio estimate ${\hat{\beta }}_{\mathrm{Rj}}$ = ${\hat{\Gamma }}_{j}/{\hat{\gamma }}_{j}$ has negligible uncertainty (so that ${\hat{\gamma }}_{j}\approx \gamma_{j}$) and can therefore be treated as a constant. This referred to as the “no measurement error (NOME)” assumption^11^, ^12^ and means that

$$w_{j}=1/\mathrm{Var}\left({\hat{\beta }}_{\mathrm{Rj}}\right)=\frac{{\hat{\gamma }}_{j}^{2}}{\mathrm{Var}\left({\hat{\Gamma }}_{j}\right)}=\frac{{\hat{\gamma }}_{j}^{2}}{\sigma_{\mathrm{Yj}}^{2}}.$$

A nonexhaustive list of GWASs with publicly available data that has been used in two‐sample summary data MR studies is given in Table 1. For a more complete list of consortia, see Haycock et al.^13^

**Table 1:** Examples of international consortia with publically available data on genetic associations with disease traits

Disease Trait	International Consortia
Alzheimer	International Genomics of Alzheimer's Project (IGAP)
Anthropometric traits	Genetic Investigation of Anthropometric Traits (GIANT)
Autism Bipolar disorder Major depressive disorder	Psychiatric Genomics Consortium (PGC)
Blood pressure	International Consortium for Blood Pressure (ICBP)
Coronary heart disease	Coronary Artery Disease Genome‐wide Replication and Meta‐analysis (CARDIOGRAM)
Glycaemic traits	Meta‐analyses of Glucose and Insulin‐related traits Consortium (MAGIC)
Lipid fractions	Global Lipids Genetics Consortium (GLGC)
Type II diabetes	Diabetes Genetics Replication and Meta‐analysis (DIAGRAM)

## 5 VISUALIZING RATIO ESTIMATES IN TWO‐SAMPLE SUMMARY DATA MR

**Figure 3:**
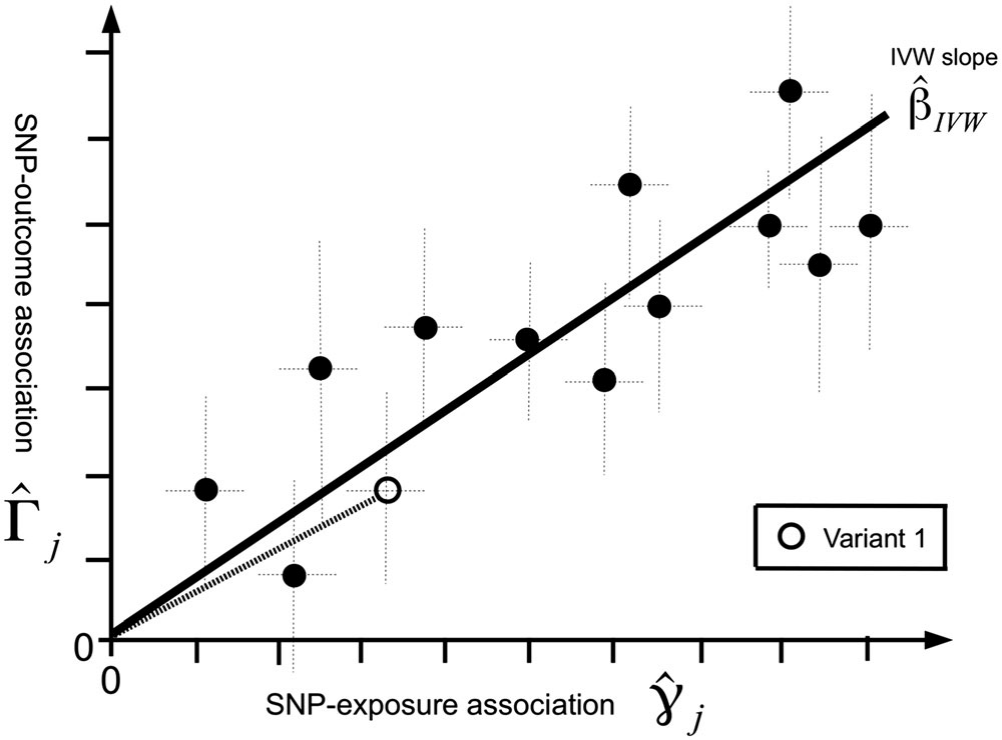
Scatter plot of single nucleotide polymorphism (SNP)‐outcome associations versus SNP‐exposure associations for a fictional Mendelian randomization (MR) analysis using 13 variants. Vertical and horizontal lines centered at each data point show 95% confidence intervals for the associations. The slope joining each data point to the origin represents the ratio estimate of a given SNP. IVW, inverse variance weighted

When conducting two‐sample summary data MR, each SNP contributes an individual ratio estimate. When inspecting the set of ratio estimates used to furnish an IVW analysis, it is standard practice to produce a scatter plot of the SNP‐outcome association estimates versus the SNP‐exposure associations. Figure 3 shows a scatter plot for a fictional MR study involving 13 ratio estimates—each dot represents an individual SNP with its association with the exposure plotted on the horizontal axis and its association with the outcome on the vertical axis. Horizontal and vertical dotted black lines indicate 95% confidence intervals for the exposure and outcome associations, respectively. By convention, the SNPs in Figure 3 have been coded so that their corresponding SNP‐exposure associations are all positive. The slope of the line joining each point to the origin is the ratio estimate for that variant, as illustrated for variant “1” in the figure. We can then interpret the IVW estimate as the slope obtained from a linear regression of the SNP‐outcome associations on the (positively oriented) SNP‐exposure associations, under the constraint that the intercept of the regression is fixed at zero. That is, the IVW estimate in (5) obtained using first order weights is identical to fitting the model:

(6) $${\hat{\Gamma }}_{j}=\beta {\hat{\gamma }}_{j}+\sigma_{\mathrm{Yj}}\epsilon_{j},\;\epsilon_{j}∼N\left(0,1\right).$$

The zero intercept constraint follows directly from the assumption that all variants are valid IVs, which in turn means that all true SNP‐exposure and SNP‐outcome association pairs satisfy Equation 1.

The two‐sample summary data framework has led to an acceleration in the implementation of MR analyses, due to its ease of implementation, and has also increased the power to detect causal associations. Unfortunately, due to the sheer number of variants that can now be easily included in such MR analyses, often with limited knowledge of their functional role, it is increasingly likely that some do not meet the IV assumptions due to a phenomenon known as horizontal *pleiotropy*.^14^ This occurs when a SNP affects the outcome through pathways other than the exposure of interest. This pathway could either be through a confounder of the exposure and outcome or via an independent pathway, as illustrated in Figure 1.

Given its growing importance and its close connection to meta‐analysis, the remainder of this review focuses on the two‐sample summary data framework, and on methods for detecting and adjusting for bias due to pleiotropy.

## 6 DETECTING HETEROGENEITY AMONG CAUSAL ESTIMATES

Valid genetic instruments should generally furnish similar estimates of causal effect. This can be easily assessed using Cochran's *Q* statistic:

(7) $$Q=\sum_{j}Q_{j}=\sum_{j}w_{j}{\left({\hat{\beta }}_{\mathrm{Rj}}-{\hat{\beta }}_{\mathrm{IVW}}\right)}^{2},$$

which is identical to the Sargan overidentification test used in econometrics.^15^ If *Q* detects substantial heterogeneity among the causal estimates, which cannot be explained by sampling variation alone, then an extension to the basic model is required. A natural way to achieve this is to include an additional term, *α*_*j*_, into Equation 1 linking the true SNP‐outcome association to the true SNP‐exposure association for SNP *j*, as in Equation 5:

(8) $$\;\Gamma_{j}={\beta \gamma }_{j}+\alpha_{j}.$$

Here, *α*_*j*_ represents the direct pleiotropic effect of SNP *j* on the outcome not through the exposure. Although horizontal pleiotropy is strictly a violation of the IV assumptions, its presence does not necessarily preclude reliable causal inference under the following two conditions:

1. Pleiotropic effects are independent in magnitude of the SNP‐exposure associations (the instrument “strength”) across all variants. This is referred to as the InSIDE assumption.^16^
2. The mean pleiotropic effect across all variants, $\bar{\alpha }$, is zero.

If both conditions hold, then the horizontal pleiotropy is said to be “balanced” and the causal effect can be reliably estimated via a standard additive or multiplicative random effects model extension to the IVW approach.^12^, ^17^, ^18^

## 7 SEPARATING WEAK INSTRUMENT BIAS FROM PLEIOTROPY

Detecting heterogeneity does not automatically imply the presence of pleiotropy. Instead, it implies that at least one of the IV assumptions, or instead one of the various modeling assumptions required specifically for two‐sample summary data MR has been violated. This could be, for example, because each SNP acts on the exposure to produce a different true causal effect. Alternatively, it could be induced by combining data on two cohorts that are not homogeneous.^12^, ^19^

A more benign factor that can lead to an easily quantifiable inflation of *Q* (even when all IV and modeling assumptions are satisfied) is when the NOME assumption used to justify first order weighting is violated because of nonnegligible uncertainty in the SNP‐exposure associations. If this is the case, the SNPs are referred to as “weak instruments,” which leads to regression dilution bias in the IVW estimate towards the null. Instrument strength for the IVW approach can be quantified using the mean *F* statistic.

$$\bar{F}=\frac{1}{L}\sum_{j=1}^{L}\frac{{\hat{\gamma }}_{j}^{2}}{\sigma_{\mathrm{Xj}}^{2}},$$

and the dilution towards zero approximated by the relation $\left(\bar{F}-1\right)/\bar{F}$.^5^, ^11^ For example, an $\bar{F}$ of 100 or 20 would indicate a likely 1% or 5% dilution in the IVW estimate, respectively. Recent work^20^ has shown that both of these negative features can be removed from the analysis by the use of more sophisticated weighting as follows: The first order weights in *Q* can be replaced with new weights of the form:

(9) $$w_{j}\left(\beta \right)=\frac{\beta^{2}\sigma_{\mathrm{Xj}}^{2}+\sigma_{\mathrm{Yj}}^{2}}{{\hat{\gamma }}_{j}^{2}},$$

where $\sigma_{\mathrm{Xj}}^{2}$ represents the variance of ${\hat{\gamma }}_{j}^{2}$ and *β* represents the causal effect parameter of interest. Next, the value of *β* is found that minimizes *Q* by setting its derivative (with respect to *β*) to zero.

The resulting minimal *Q* statistic is free from inflation due to weak instruments. The optimized value of *β* flowing from the use of the weights in (9) is an improved IVW estimate that is free from regression dilution bias under a fixed effect model. However, if heterogeneity is detected by the minimal *Q* statistic, then new weights that incorporate an additional random effects heterogeneity parameter must be defined before the *Q* statistic is minimized with respect to both parameters. For example, under an additive random effect model, this weight function would be

(10) $$w_{j}\left(\beta ,\tau^{2}\right)=\frac{\beta^{2}\sigma_{\mathrm{Xj}}^{2}+\sigma_{\mathrm{Yj}}^{2}+\tau^{2}}{{\hat{\gamma }}_{j}^{2}},$$

with *τ*^2^ representing the additional variance due to pleiotropy. For further technical details including a multiplicative random effects model implementation, see other studies.^5^, ^20^

## 8 ACCOUNTING FOR HETEROGENEITY AND BIAS DUE TO DIRECTIONAL PLEIOTROPY

Pleiotropy is said to have a directional element when the mean pleiotropic effect across all variants, $\bar{\alpha }$, is nonzero, which induces bias in the standard IVW estimate. Directional pleiotropy can be viewed as analogous to the phenomenon of “small study bias” that affects mainstream meta‐analyses of published study results. That is, a trend in study effect estimates according to their sample size. Small study effects can be formally tested for by the presence of a nonzero intercept in a regression of study estimates on their standard errors. This is the principle of Egger regression.^21^ In the MR context, directional pleiotropy can be assessed by performing “MR‐Egger regression.”^16^ This is a meta‐regression of SNP‐outcome association estimates on the corresponding SNP‐exposure association estimate, after they have been oriented in the positive direction. This is identical to the standard IVW approach, except that the intercept of the regression slope is estimated, rather than being fixed to zero. For example, if first order weights are used as in (6), then the MR‐Egger model would be

(11) $${\hat{\Gamma }}_{j}=\beta_{0}+\beta {\hat{\gamma }}_{j}+\sigma_{\mathrm{Yj}}\epsilon_{j},\;\epsilon_{j}∼N\left(0,1\right).$$

Providing that the InSIDE assumption holds and the SNP‐exposure associations are precise enough for first order weighting to be appropriate, testing for a nonzero intercept in MR‐Egger regression is then equivalent to testing for directional pleiotropy, and the MR‐Egger slope provides a consistent estimate of the causal effect.^16^

**Figure 4:**
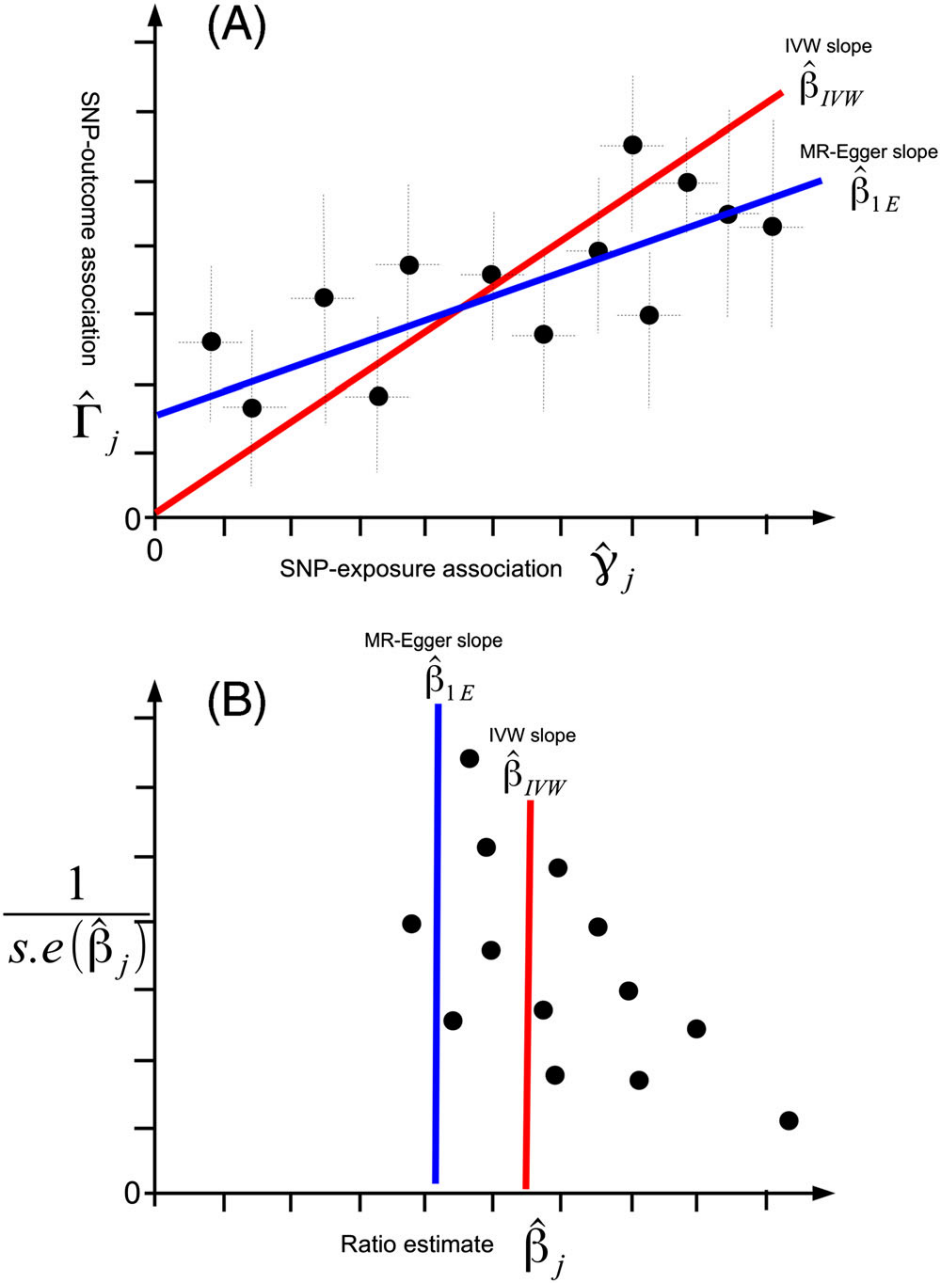
A, Hypothetical scatter plot with directional pleiotropy. Consequently, MR‐Egger estimates a nonzero intercept. B, Hypothetical funnel plot. Directional pleiotropy is seen to induce asymmetry. The MR‐Egger estimate can be interpreted as the value that would have been obtained if the funnel plot were symmetrical.

The presence of directional pleiotropy can be visually assessed using a scatter plot, of SNP‐outcome associations versus SNP‐exposure associations, as illustrated for hypothetical data in Figure 4A. It can also be assessed using a funnel plot^16^ (as shown for the same hypothetical data in Figure 4B), which displays causal effect estimates on the horizontal axis versus their square root precision on the vertical axis. When there is no pleiotropy or balanced pleiotropy and the InSIDE assumption holds, then:

- The intercept of the MR‐Egger regression model will not differ substantially from zero.
- The funnel plot should appear symmetrical, in that less precise estimates should funnel in from either side towards the most precise estimates.
- The IVW and MR‐Egger causal estimates will be consistent with each other.

## 9 THE PERFORMANCE OF MR‐EGGER REGRESSION IN PRACTICE

### 9.1 When does MR‐Egger provide a better fit to the data?

The utility of applying MR‐Egger regression in a given scenario can be assessed by seeing how much of the heterogeneity about the IVW estimates can be successfully explained by the addition of an intercept term in the model. As a basic principle, MR‐Egger will explain more heterogeneity as the magnitude of its intercept increases (away from the null). This can be explicitly quantified using an extension to Cochran's *Q* statistic—namely, Rucker's *Q*^′^ 12, 22—that was proposed for use in mainstream meta‐analysis. When first order weights are used, *Q*^′^ can be written as follows:

(12) $$Q^{\prime }=\sum_{j=1}^{L}w_{j}{\left({\hat{\beta }}_{\mathrm{Rj}}-\frac{{\hat{\beta }}_{0}}{{\hat{\gamma }}_{j}}-\hat{\beta }\right)}^{2},$$

where ${\hat{\beta }}_{0}$ and $\hat{\beta }$ are the MR‐Egger estimates obtained from model (11). To formally test whether the MR‐Egger model provides a better fit, the difference *Q* − *Q*^′^ can be compared with a $\chi_{1}^{2}$ distribution.^12^

### 9.2 Precision and weak instrument bias of MR‐Egger regression

In practice, the IVW estimate is likely to yield far more precise estimates than MR‐Egger regression. An important factor affecting only the precision of MR‐Egger is the amount of variation between the set of SNP‐exposure associations, once they have been oriented in the positive direction. That is, it works best when there are SNPs with both small, medium, and large associations relative to one another. This is true when fitting any sort of univariable linear regression model with an intercept because the explanatory variable of the regression (in this case ${\hat{\gamma }}_{j}$) must exhibit some variation, the more the better. When such variation is not present over and above what would be expected by the SNP‐exposure association standard errors, *σ*_*Xj*_, (as represented by the horizontal error bars in Figure 4A), it would suffer complete regression dilution bias. That is, its estimate would be shrunk on average to zero. Rather being used in its original guise to quantify heterogeneity among causal estimates, Higgins *I*^2^ statistic^23^ has been repurposed in MR to quantify the expected dilution of MR‐Egger regression estimates, by calculating it with respect to the SNP‐exposure summary data $\left({\hat{\gamma }}_{j},\sigma_{\mathrm{Xj}}^{2}\right)$.^11^ This is referred to as “ $I_{\mathrm{GX}}^{2}$.” An $I_{\mathrm{GX}}^{2}$ close to 1 would indicate no dilution, whereas an $I_{\mathrm{GX}}^{2}$ of 0.5 would indicate a likely 50% dilution. Note that an $I_{\mathrm{GX}}^{2}$ of 1 is equivalent to the NOME assumption being satisfied. This could be achieved even if there were very little variation between the SNP‐exposure association estimates, as long as they are very precise. $I_{\mathrm{GX}}^{2}$ is therefore a measure of the collective strength of a set of instruments for MR‐Egger. The errors‐in‐variables technique of simulation extrapolation has successfully been applied to correct for this weak instrument bias when $I_{\mathrm{GX}}^{2}$ is sufficiently low.^11^ Further research is ongoing to extend the modified weights in Equation 10, so that they can be used for both IVW and in MR‐Egger regression.

Because of its relative imprecision, MR‐Egger regression is not advocated to replace the standard IVW approach. Indeed, it is best utilized within the context of a sensitivity analysis,^14^, ^24^ and given most credence when it provides a demonstratively better fit to the data.^12^

### 9.3 Robust meta‐analytic approaches

The InSIDE assumption is likely to be violated when a SNP is associated with the exposure of interest through a confounder of the exposure‐outcome relationship (as represented by the dotted arrow between *G* and *U* in Figure 1). This is because it would make the magnitude of an SNP's pleiotropy correlated with its strength as an instrument.^12^ This invalidates both the IVW and MR‐Egger analyses. For this reason, robust meta‐analytic methods have been proposed,^25^, ^26^ which do not rely on the InSIDE assumption, and are being increasingly implemented alongside IVW and MR‐Egger. Specifically, rather than calculating an IVW mean of all ratio estimates (eg, the IVW estimate):

- The “weighted median” estimate^25^ calculates median of the IVW empirical distribution function of ratio estimates.
- The mode‐based estimate (MBE)^26^ calculates the modal value of the same weighted empirical distribution function.

Currently, both approaches use first order inverse variance weights to define their empirical distribution functions. The weighted median can provide a consistent estimate for the causal effect even if up to half of the SNPs violate InSIDE (ie, most SNPs do not). The MBE can provide a consistent estimate if valid SNPs (ie, those with a zero value of *α*_*j*_ in Equation 5) form the largest subset of all SNPs that have the same value of *α*_*j*_.

In order to improve the robustness of IVW and MR‐Egger regression, outlier detection and removal strategies have also been proposed. For example, in Bowden et al,^20^ the individual contribution of each SNP to Cochran *Q* can be assessed informally against a $\chi_{1}^{2}$ distribution to see whether a small number of SNPs are driving the apparent heterogeneity and are therefore candidates for removal in a sensitivity analysis. This approach will be demonstrated in the applied example below. Studentized residuals and Cook's distance have also been used in MR studies to detect influential SNPs^27^ that merit closer inspection. The Galbraith radial plot has additionally been repurposed for detecting outlying variants in MR.^28^

### 9.4 Example: examining the causal effect of SBP on CHD

We illustrate the methods described thus far by reanalyzing a two‐sample summary data MR study previously reported by Ference et al^29^ and Bowden et al^20^ that examined the causal effect of systolic blood pressure (SBP; the exposure) on CHD (the outcome). SNP‐exposure association estimates were obtained from the International Consortium for Blood Pressure (ICBP) GWAS consortium for 26 variants that were robustly associated with SBP at genome‐wide statistical significance levels. Log‐odds ratio estimates of SNP‐CHD association were collected from Coronary Artery Disease Genome‐wide Replication and Meta‐analysis (CARDIOGRAM) consortium. Both data sources are publically accessible (see Table 1); however, we provide these data as Supporting Information for the interested reader.

**Figure 5:**
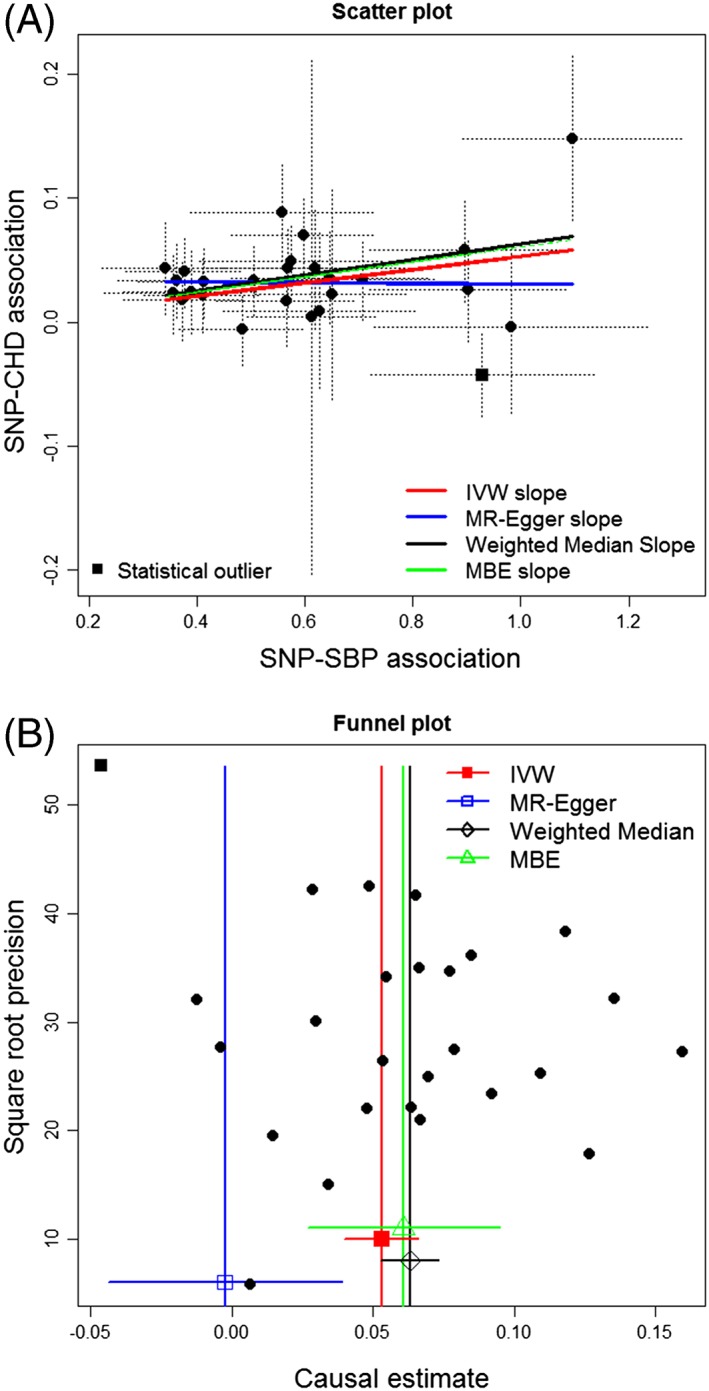
A, Scatter plot of the SBP data horizontal and vertical dashed lines show 95% confidence interval for each association. IVW, MR‐Egger, weighted median slope, and MBE slope are also shown. B, Funnel plot of the SBP data. Data on variant rs17249754 are represented by a square in each plot. CHD, coronary heart disease; IVW, inverse variance weighted; MBE, mode‐based estimate; MR, Mendelian randomization; SNP, single nucleotide polymorphism

Figure 5 shows a scatter plot of the SNP‐CHD versus the SNP‐SBP associations along with their 95% confidence intervals and its corresponding funnel plot. Causal effect estimates for the log‐odds ratio of CHD for a 1 mmHg increase in SBP were obtained via the IVW and MR‐Egger approaches. Estimates for the weighted median estimator and MBE are also shown. The SNP‐exposure association estimates were sufficiently precise (a mean *F* statistic of 61) and sufficiently varied (an $I_{\mathrm{GX}}^{2}$ statistic of 0.96) for the NOME assumption to approximately hold. We therefore use first order weights for all estimators in the analysis. Full results are given in Table 2. To improve their clinical relevance, the estimates in Table 2 are shown as odds ratios and reflect the effect of a 5 mmHg increase in SBP.

**Table 2:** Results for the SBP‐CHD MR analysis. Estimates reflect the odds ratio for a 5 mmHg increase in SBP on the risk of CHD. Results shown for all methods with and without variant rs17249754

Method (All Variants)	OR (95% CI)	*P* Value	Heterogeneity Statistic (*P* Value)
IVW	(Slope) 1.30 (1.18–1.44)	3.01 × 10 ^−5^	*Q* = 67.1 (1.03 × 10 ^−5^ )
MR‐Egger	(Intercept) 1.18 (0.99–1.41)	0.0745	*Q*′ = 58.6 (1.00 × 10 ^−4^ )
	(Slope) 0.99 (0.73–1.34)	0.934	
Weighted median	(Slope) 1.37 (1.23–1.52)	1.57 × 10 ^−6^	*Q* − *Q′* = 8.5 (3.60 × 10^−3^)
MBE	(Slope) 1.35 (1.13–1.61)	0.002	

Method (rs17249754 Removed)	OR (95% CI)	*P* Value	Heterogeneity Statistic
IVW	(Slope) 1.39 (1.28–1.51)	2.63 × 10 ^−8^	*Q* = 35.0 (0.068)
MR‐Egger	(Intercept) 1.05 (0.91–1.22)	0.51	*Q*′ = 34.3 (0.061)
	(Slope) 1.27 (0.97–1.67)	0.09	*Q* − *Q*′ = 0.7 (0.4)
Weighted median	(Slope) 1.39 (1.25–1.53)	1.25 × 10 ^−6^	
MBE	(Slope) 1.36 (1.14–1.61)	0.002	

Abbreviations: IVW, inverse variance weighted; MBE, mode‐based estimate; MR, Mendelian randomization.

The IVW, weighted median, and MBE approaches all suggest a positive causal effect of SBP on CHD. MR‐Egger regression, by contrast, infers that directional pleiotropy is largely driving the analysis and suggests a causal effect close to zero.

We would expect *Q* and *Q′* statistics to be equal to their degrees of freedom (25 and 24, respectively), under the null hypothesis of no heterogeneity. Since they are both more than twice this value, substantial heterogeneity around the IVW and MR‐Egger estimates is detected that could be due to horizontal pleiotropy. The difference *Q* − *Q*^′^ = 8.5 is extreme under a $\chi_{1}^{2}$ distribution, which suggests that MR‐Egger is a better fit to the data. A more detailed outlier analysis revealed that this heterogeneity was largely driven by a single outlying variant—rs17249754 in the *ATP2B1* gene (shown as a square rather than a circle in Figure 5). It alone contributes a value of 28.3 to *Q*, which equates to 42% of its total. The next largest individual SNP contribution is 8.4. Since rs17249754 is a relatively strong and potentially pleiotropic instrument in the analysis, this could lead to the InSIDE assumption being violated, and be responsible for the large discrepancy between the IVW and MR‐Egger results. Repeating the analysis after the removal of rs17249754 shows the estimates are indeed in broad agreement (Table 2), and statistical heterogeneity around the IVW and MR‐Egger estimates is substantially reduced (as noted by the values of *Q* and *Q′*). Furthermore, the difference *Q* − *Q*^′^ = 0.7 now indicates that MR‐Egger does not provide a substantially better fit to the data. The weighted median and MBE results are least affected by the removal of rs17249754, highlighting their inherent robustness to outliers.

In summary, our MR analysis supports the hypothesis that SBP is causally related to CHD risk, which aligns these findings to meta‐analysis of equivalent trial evidence.^30^

## 10 CONCLUSIONS AND FUTURE DIRECTIONS

Meta‐analysis methods have been used in MR investigations throughout its short lifetime, initially as a tool for aiding collaborative analysis of individual level data across epidemiological studies and latterly for synthesizing GWAS results within two‐sample summary data MR. Established techniques for detecting heterogeneity and bias in meta‐analysis have successfully been applied to MR to both test and adjust for violations of the IV assumptions. However, the direction of methodology is not just one way: MR‐Egger regression has recently been proposed as a means to adjust the analysis of multicenter randomized trials for nonadherence^31^ and to examine the mechanism of action for statins.^32^ Median‐ and mode‐based estimation have also been suggested as sensitivity analysis tools for meta‐analyses of RCTs with suspected small study effects.^33^

Our description of the summary data MR approaches in this paper assume that the SNPs used in the analysis are sufficiently separated in the genome so as to be mutually uncorrelated. This justifies the use of standard weighted least squares to estimate the parameters in IVW and MR‐Egger regression and also underlies the simple empirical density functions used by the weighted median and MBE. Both IVW and MR‐Egger regression can easily be extended to the case of correlated variants. In that case, the model parameters must be estimated using generalized least squares by specifying a correlation matrix for the set of SNPs.^34^ Extensions for the weighted median and MBE for correlated variants have yet to be explored and is an interesting avenue for further research.

Summary data MR analysis relies on obtaining SNP‐trait associations from a GWAS, which is itself usually a conglomeration of data from many studies. Meta‐analysis is therefore required to derive the necessary estimates. Fixed effect models are typically used for this purpose,^35^ for example, the most widely used software package METAL^36^ does not have a random effects option. If heterogeneous results are obtained for a single SNP across studies, whole studies are sometimes removed to promote the fixed effect analysis. State‐of‐the‐art methods for random effects meta‐analysis^37^, ^38^ might have considerable utility in improving the summary information flowing from a GWAS, which would then affect subsequent summary data MR analyses. This is another area for future research.

The uptake and implementation of two‐sample summary data MR is being facilitated by software packages in R^39^, ^40^ and Stata^41^ to implement all of the analysis methods highlighted in this paper, and more. In particular, MR‐BASE (http://www.mrbase.org/)^40^ is an analytical web‐based platform linking genetic and trait summary data from over 1000 studies and 14 million samples with state‐of‐the‐art tools for MR analysis. This has enabled causal relationships to be assessed with ease on an unprecedented breadth and scale. In time, it may be necessary for analysis and reporting guidelines, which have worked successfully for meta‐analyses of clinical trials,^42^ to be agreed on to help ensure that MR analyses remain a principled and reliable means to probe causal questions in the new era of “big data.”

## CONFLICTS OF INTEREST

The author reported no conflict of interest.

## Supporting information

Data S1. Supporting information
